# A review of the HIV/AIDS situation in northeastern Nigeria

**DOI:** 10.1186/1742-4690-8-S2-P86

**Published:** 2011-10-03

**Authors:** Musa A Garbati, Abdullah A Abba, Danjuma N Kabrang, Haruna Yusuph

**Affiliations:** 1 Division of Infectious Diseases, Department of Medicine, King Fahad Medical City, Riyadh, Saudi Arabia; 2 Department of Medicine, ABU Zaria, Nigeria; 3 FHI-GHAIN Bauchi Zonal Office, Nigeria; 4 College of Medicine, University of Maiduguri, Nigeria

## Introduction

The HIV/AIDS situation in Nigeria has undergone a lot of transformation after a long period of denial. Sero Prevalence Sentinel Surveys were conducted among antenatal clinic (ANC) attendees considered to be a homogenous community of persons with steady sexual partners.

## Method

The study population was made up of 36,427 consecutive pregnant women, aged 15–49 years, attending antenatal clinics in 160 selected sites across the 36 States and the FCT of Nigeria.

## Results

Fifty eight percent of the attendees were aged 20 to 29 years with the least population (2.4%) aged 40-49 years. Most of the women were married (96.4%) and this makes the population fairly homogenous in all the zones. The study found that HIV prevalence was highest in NC Zone (7.5%) followed by SS Zone (6.5%). The NW Zone had the lowest prevalence of 2.1%. The epidemic has grown beyond the high-risk groups (in which it was earlier described) to affect the general population.

Based on these figures, an estimated 3.1 million people are living with HIV/AIDS in Nigeria in 2010, harboring the second highest number of people living with HIV (PLWHA) in the world, second only to South Africa (UNAIDS HIV epidemic update 2010). Although HIV prevalence is much lower in Nigeria than in other African countries, the size of Nigeria’s population means that the disease burden is much higher (UNAIDS 2010 'UNAIDS report on the global AIDS epidemic').

Prevalence rates were higher in urban areas than rural areas. The highest site prevalence of 21.3% in the country was reported in Wannune (Benue State) while the lowest prevalence of 0.0% was reported in four sites, namely Kwami (Gombe State), Rano (Kano State) Owhelogbo (Delta State) and Ganawuri (Plateau State). The HIV prevalence rate for the six states of the Northeast is shown in table 1 below.

**Table 1:** HIV prevalence (%) in the six states of northeastern Nigeria from 1991 to 2010

*STATE*	‘91/’92	‘93/’94	‘95/’96	1999	2001	2003	2005	2008	2010
*ADAMAWA*	*0.3*	*1.3*	*5.3*	*5.0*	*4.5*	*7.6*	*4.2*	*6.8*	*3.8*
*BAUCHI*	*ND*	*ND*	*ND*	*3.0*	*6.8*	*4.8*	*3.4*	*3.1*	*2.0*
*BORNO*	*4.4*	*6.4*	*1.0*	*4.5*	*4.5*	*3.2*	*3.6*	*2.0*	*5.6*
*GOMBE*	*ND*	*ND*	*ND*	*4.7*	*8.2*	*6.8*	*4.9*	*4.0*	*4.2*
*TARABA*	*ND*	*ND*	*6.0*	*5.5*	*6.2*	*6.0*	*6.1*	*5.2*	*5.8*
*YOBE*	*ND*	*ND*	*ND*	*1.9*	*3.5*	*3.8*	*3.7*	*2.7*	*2.1*

## Conclusion

The results from the latest survey indicate that the trend is falling. However, prevalence remains relatively high for some regions. Infection burden has varied between rural and urban areas as reported previously. HIV is a "social disease". It disrupts the fabrics of society through stigmatization of sufferers, as well as through years of education and of productivity lost. In the absence of an effective vaccine, education has been described as a “social vaccine” in the fight against the pandemic. Governments have to act in concert with non-governmental agencies to help this region of Nigeria and others attain at least the first six of the millennium development goals (MDGs) by the year 2015 to check this ugly trend.

